# Targeting *Trypanosoma cruzi* with silver and gold-based N-heterocyclic carbene complexes: insights into parasite death and trypanothione reductase interaction

**DOI:** 10.1007/s10534-025-00731-4

**Published:** 2025-08-14

**Authors:** Yuly Bernal, Angie Melo Marquez, Hector Rafael Rangel, Maria Cristina Goite, Pedro Noguera, Franmerly Fuentes, Rubén Machado, William Castro, Vaneza Paola Lorett Velasquéz, Cristian Buendia-Atencio, Eduvan Valencia Cristancho, Anny Karely Rodriguez, Silvio Lopez-Pazos, Monica Losada-Barragán

**Affiliations:** 1 Facultad de Ciencias, Universidad Antonio Nariño-Sede Circunvalar, Cra. 3 Este # 47A - 15, Bogotá, D.C. Colombia; 2 Laboratorio de Virología Molecular, Venezuelan Institute for Scientific Research, IVIC, Caracas, Venezuela; 3 Centro de Química, Instituto Venezolano de Investigaciones Científicas (IVIC), Caracas, 1020-A Venezuela; 4 Facultad de Medicina y Ciencias de La Salud, Universidad Militar Nueva Granada, Bogotá, D.C. Colombia; 5 Área de Ciencias Naturales, Secretaría de Educación Distrital de Bogotá, Bogotá, D.C. Colombia

**Keywords:** *Trypanosoma cruzi*, Carbenic derivative, ROS, Trypanothione reductase

## Abstract

Chagas disease remains a major public health challenge, and there is a need for new therapeutic agents. N-heterocyclic carbene (NHC) complexes, particularly those linked to silver or gold, have shown significant anticancer, antimicrobial, and antiparasitic activities. This study aimed to evaluate the efficacy of four NHC compounds (QMT3, QMT4, QMT7, and QMT8) against *Trypanosoma cruzi*, the causative agent of Chagas disease. In vitro assays revealed that QMT3 and QMT8 exhibited the strongest antiparasitic effects, with QMT3 showing the highest potency and stability over time (IC₅₀ = 10.3 µg/mL at 24 h). Both compounds induced rapid, irreversible cell death in epimastigotes, primarily through late apoptotic-like and necrotic pathways, as evidenced by Annexin V/PI labeling. Additionally, treatment with QMT3 and QMT8 led to significant increases in intracellular reactive oxygen species (ROS), particularly superoxide (SO). Notably, both compounds displayed high specificity for the parasite with low cytotoxicity towards mammalian cells, although QMT8 was less toxic to host cells than QMT3 at short exposure times. Molecular modeling studies revealed that QMT3, and QMT8 bind to the active site of TryR, a crucial player in maintaining redox homeostasis in trypanosomatids, potentially competing with its natural ligand and disrupting its enzymatic function. These findings suggest that QMT3 and QMT8, silver- and gold-based NHC complexes, act through redox system disruption and TryR inhibition, positioning them as promising candidates for the development of new treatments for Chagas disease.

**Supplementary Information:**

The online version contains supplementary material available at 10.1007/s10534-025-00731-4.

## Introduction

Chagas disease, or American human trypanosomiasis, is a neglected infectious disease caused by *Trypanosoma cruzi* (subgenus Schizotrypanum). This parasite is distributed throughout the Americas, particularly in Central and South America, affecting approximately 6–8 million people, mainly among low-income populations (Desquesnes et al. 2022). It is primarily transmitted by triatomine bugs, though it can also spread via consumption of contaminated food and congenital routes (Fonseca-Berzal et al. 2016).

Chagas disease can be asymptomatic or present mild, unspecific symptoms during the acute phase. However, in the chronic phase, it can lead to serious clinical manifestations, including cardiac, neurological, or gastrointestinal complications that can be life-threatening (WH Organization 2024). Prevention strategies focus on vector control to reduce the spread of the parasite, detection of infected pregnant women, and improving quality control in blood and organ banks (Liu and Zhou 2015; Rivera et al. 2023). However, challenges remain, such as insecticide resistance, lack of consensus on the best treatment for chronic infections, limited availability of current treatment drugs, absence of an agreed standard of diagnosis, and no available vaccine (Reithinger et al. 2009).

The preferred treatments for Chagas disease are nifurtimox and benznidazole, which are generally effective in the acute and early chronic phases. However, side effects are common with both drugs. The most frequent side effects associated with nifurtimox include anorexia, weight loss, mental health issues, and gastrointestinal problems. Benznidazole primarily causes skin reactions and may also result in serious complications, such as bone marrow suppression, thrombocytopenic purpura, and agranulocytosis (Castro et al. 2006). Both drugs negatively impact the adrenal glands, colon, and esophagus and are mutagenic (Castro et al. 2006; Perez-Molina et al. 2021).

N-heterocyclic carbene (NHC) compounds are a class of ligands widely utilized in reactions catalyzed by transition metal complexes, such as gold and silver. The functionalization of these molecules is possible due to the reactivity of nitrogen in the heterocyclic rings, which leads to the precursor carbenic carbon (Jacobsen et al. 2009). NHC compounds have shown to be effective carrier ligands for metals in complexes, allowing them to pass through cell membranes or maintain stability in solution, thereby facilitating interactions with biological targets (Melaiye et al. 2004). Also, NHCs would probably exhibit lower toxicity than synthetic drugs; hence, they have been investigated for treating infectious diseases (Akkoç et al. 2016; Gök et al. 2014). Metals of group 11, like gold or silver, have been used for medicinal purposes since ancient times and recently as antirheumatic, antimicrobial, or antitumoral drugs (de Paiva et al. 2020). Specifically, the complexes [Ag(C_13_H_13_N_5_)]_2_Br_2_] and [Au_2_(C_13_H_13_N_5_)Cl_2_] have demonstrated significant in vitro activity against HIV-1, inhibiting viral replication by 55% and 82%, respectively (Winter et al. 2017). Also, other NHC compounds have shown efficacy against some parasite species of the genus *Leishmania infantum* (Zhang et al. 2018) and *T. brucei* (Winter et al. 2017).

Gold and silver complexes have been shown to inhibit the activity of the thioredoxin reductase (TrxR) system, which regulates the cellular redox state (Berners-Price and Filipovska 2011; Sánchez et al. 2016). The proposed mechanism of inhibition by gold complexes involves the formation of covalent bonds between the metal and selenium atoms in the enzyme, resulting in alterations to its three-dimensional (3D) structure (Nobili et al. 2010). Consequently, the potential mechanism of NHC derivatives may be related to this system.

Trypanothione reductase (TryR) is a flavoenzyme that relies on NADPH for its activity, maintaining the balance between trypanothione disulfide (TS2) and trypanothione dithiol (T(SH)2) by reducing TS2. The synthesis of trypanothione occurs from one molecule of spermidine and two molecules of glutathione, which is characteristic of trypanosomatid metabolism. TryR provides a protective mechanism for the parasite against oxidative stress from free radicals, such as hydroxyl radicals (OH·), superoxide (O_2_^−^), and hydrogen peroxide (H_2_O_2_), making it a promising molecular target (Garrard et al. 2000).

This work evaluated NHC derivatives under in vitro conditions for their antiparasitic action against *T. cruzi*. Additionally, the potential interaction of the complexes with TryR was assessed using molecular docking to generate targeted interactions between selected compounds, acting as ligands, and the active sites of the enzyme or other relevant regions.

## Materials and methods

### Carbene synthesis

All syntheses were carried out as described in Supplementary Information under an inert atmosphere and without light. The solvents were previously dried and distilled before using standard methods (Armarego and Perrin 1988). The ligands 1-allyl-3methylbenzimidazolin-2-yliden-1yl) bromide and 2,6-bis(3-methylimidazolin-2-yliden-1-yl)pyridine dibromide and its derivatives Bis(1-allyl-3methylbenzimidazolium)silver(I) hexafluorophosphate (QMT3) (599,30 g/mol), Bis(1-allyl-3methylbenzimidazolium)gold(I) hexafluorophosphate (QMT4) (688.39 g/mol), Bis(1,1 ´-(2,6-pyridyl)-3,3́- dimethyldiimidazolium)disilver(I)bromide (QMT7) (693.17 g/mol) and (1,1 ´-(2,6 pyridyl)-3,3 ´-dimethyldiimidazolium)dioro(I) bromide (QMT8) (704.02 g/mol) were prepared according to corresponding reports (Chen and Lina 2000; Ghdhayeb et al. 2014; Sánchez et al. 2016). The carbenes were resuspended in DMSO, and for each test, were diluted in culture medium.

**Fig. 1:**
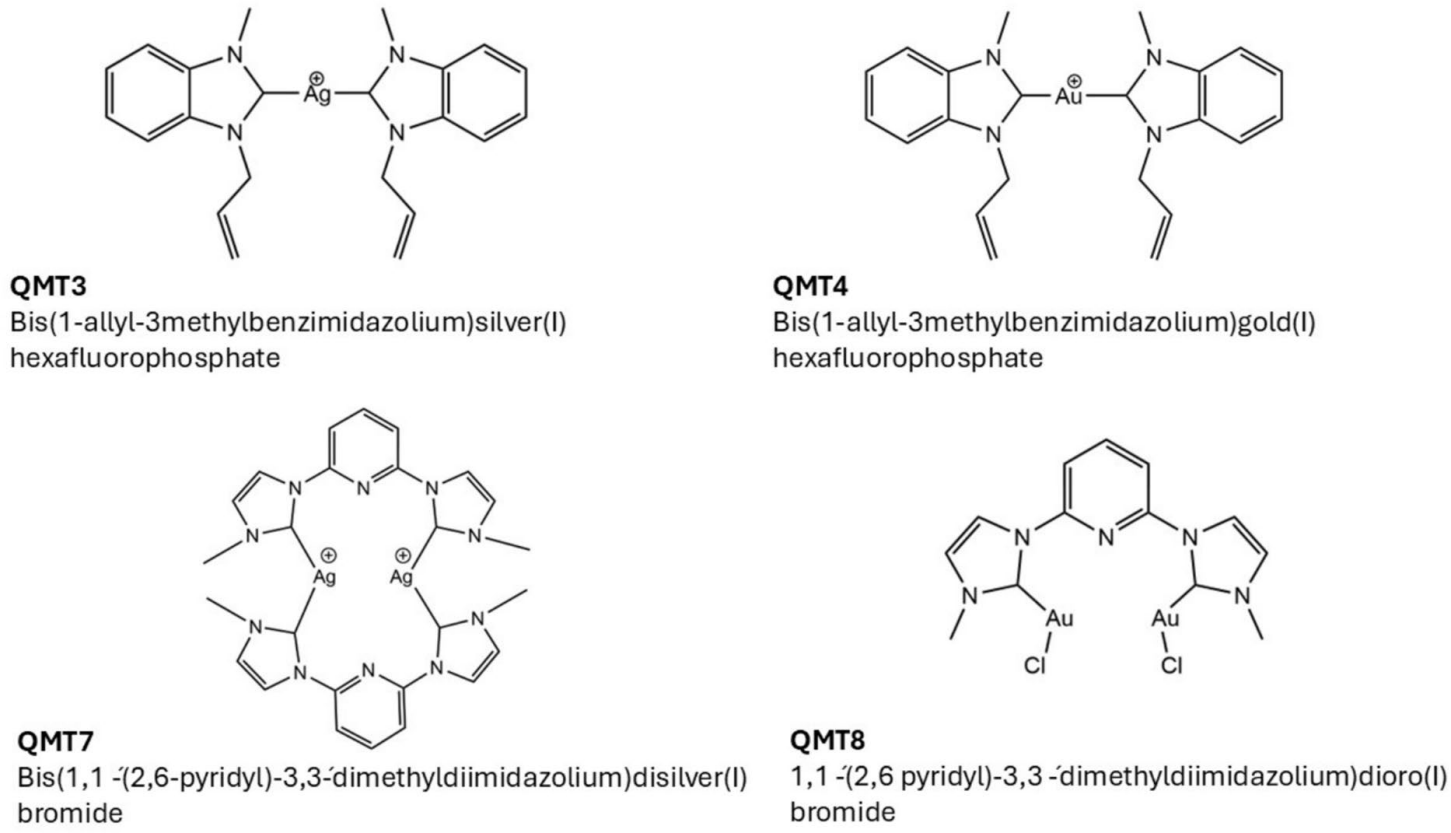
Synthesized N-heterocyclic carbene derivatives

The four synthesized complexes are shown in Fig. 1. These are neither commercial nor derived from natural products, so their structures are not found in conventional databases.

### Culture of parasites

The epimastigote forms of *T. cruzi* clone Y were sustained through weekly transfers in liver infusion tryptose (LIT) medium supplemented with 10% heat-inactivated fetal bovine serum (FBS) at a temperature of 28 °C (Batista et al. 2015). The *T. cruzi* parasites of the Y strain utilized in this study were sourced from our laboratory stock.

### Susceptibility assay

The experiment was conducted in 96-well microtiter plates, with each well receiving 300 μL of a suspension in LIT containing 5 × 10^6^ parasites mL^−1^. Epimastigotes were cultured at 28 °C and exposed to varying concentrations of QMT3, QMT4, QMT7, or QMT8 (200, 100, 50, 25, 12,5 6.2 and 3,1 μg/mL) and their viability was monitored daily up to 72 h. Each assay included triplicate samples. Untreated parasites were used as the negative control, while 96% dimethyl sulfoxide (DMSO) served as the positive control for inducing cell death. After each time point, parasite mobility and survival were assessed using light microscopy in a Neubauer chamber. The viability of the epimastigotes was evaluated with the trypan blue dye exclusion method. The experiments were independently conducted under the same conditions (n = 3), and the IC_50_ (concentration causing 50% parasite death) was determined by plotting compound drug concentrations against percentages of parasite death (%PD). The results are presented as the mean IC_50_ value ± standard deviation (S.D.) (Fonseca-Berzal et al. 2016).

### Flow cytometry analysis

A concentration of 5 × 10^6^ epimastigotes/mL in LIT undergoes treatment for 24 h at 28 °C using the respective IC_50_/24 h concentrations of compounds QMT3 and QMT8. Untreated parasites were used as the negative control, while 96% dimethyl sulfoxide (DMSO) served as the positive control for inducing cell death.

Epimastigotes samples were labeled for Annexin V-FITC and PI according to the manufacturer instructions (Elabscience). Live cells do not stain with either Annexin V or PI. Cells in early apoptosis are positive for Annexin V but negative for PI, while cells in late apoptosis or necrosis are positive for both Annexin V and PI. Data acquisition and analysis were conducted using a BD Accuri™ C6 flow cytometer (BD Biosciences). 10,000 events were recorded within the predefined region corresponding to epimastigotes.

### Cytotoxicity assays

Vero cells (ATCC CCL-81) were cultured in 25cm^2^ cell culture flasks with DMEM medium supplemented with 5% FBS at 37 °C in a humidified 5% CO_2_ atmosphere (Batista et al. 2015). The toxic effects of the compounds QMT3, QMT4, QMT7 and QMT8 (200 µg/mL to 6,25 μg/mL), on non-infected mammalian cells were assessed by exposure in Vero Cells for 48 h. Follow-up was conducted every 24 h. The viable cells were quantified using the MTT method (Salomão et al. 2010).

### ROS and SO evaluation

Epimastigotes of *T. cruzi* (1 × 10⁶ cells/mL) were exposed to the IC₅₀ concentrations of QMT-3 and QMT-8 for 24 h. After incubation, the cultures were centrifuged at 2500 rpm for 10 min, and the supernatants were carefully discarded. The resulting cell pellets were resuspended in fresh medium to a final volume of 500 µL. An equal volume (500 µL) of 2X ROS/Superoxide (SO) Detection Solution was then added to each sample.

For the negative control, cells were pretreated with N-acetyl-L-cysteine (NAC), a known ROS inhibitor, for 30 min prior to staining. Pyocyanin was used as a positive control to induce ROS production in mammalian cells and menadione in *T. cruzi*. All samples were incubated in the dark at room temperature for 30 min with gentle agitation at regular intervals. No additional washing steps were required prior to flow cytometric analysis. Intracellular ROS and SO levels were subsequently assessed by flow cytometry.

### Statistical analysis

Statistical analysis was conducted using Student’s t-test and one-way ANOVA test (p < 0.05) via GraphPad Prism Software version 8.4.0. Each experiment was repeated at least three independent times, including three technical replicates.

### Molecular docking

The crystal structure of the enzyme TryR (PDB ID: 1AOG) was obtained from the Protein Data Bank (PDB) database, coming from the organism *T. cruzi* with a resolution of 2.3 Å. The molecule preparation was carried out in UCSF Chimera 1.17.3 from the.pdb file, as it is a homodimeric enzyme that has an active center in both subunits, one of the chains of the protein (B) is eliminated and work was done with chain A, others cocrystallized molecules with the protein were filtered, including the coenzyme flavin adenine dinucleotide (FAD) present in both chains of the protein and maleic acid (MAE) present only in chain A, used during the crystallization of TryR. Additionally, the ions that may be present are cleaned, as well as water molecules (H_2_O), and the polar hydrogens and Kollman charges are added in order to assign partial charges to the amino acids that require it. Finally, the obtained structure was saved as a. pdbqt file for molecular docking.

3D optimization of the ligands was carried out with Avogadro Software 1.2.0 (Hanwell et al. 2012), the hydrogens were added at pH = 7.0, and then an energy minimization was performed using the UFF force fields since the molecules contain metals such as Ag and Au. UFF allows for general parameterization of metallic atoms. However, this approach represents a simplification of the actual coordination chemistry of metal complexes. Therefore, it is acknowledged that the optimized geometry may not fully reflect the specific electronic characteristics of the metal’s coordination environment. Subsequently, the ligands were processed with AutoDockTools (ADT) version 1.5.7 (Sanner 1999), where newly all the hydrogens were added, the Gasteiger charges were computed, and the non-polar hydrogens were applied.

Interaction energies were obtained using ADT, followed by visualization with relevant software. Additionally, using manual docking, the parameterization was performed on the input file to correct administered electrostatic information necessary for recognizing atoms that are not standardized, in this case, the metals Au and Ag [https://github.com/marekolsak/fastgrid/tree/master/autogrid_original_4_2_1_ref/autodock].

**Fig. 2:**
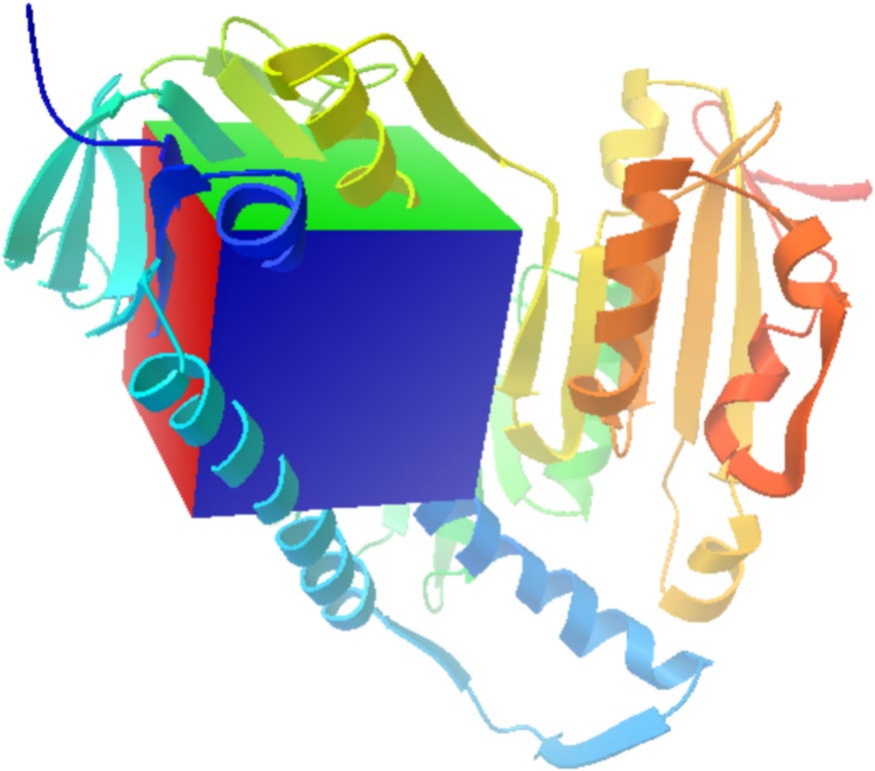
Grid box of dimensions 60 × 60 × 60 Å used in the molecular docking of the selected compounds and the enzyme TryR

The previously prepared protein was exported to the program as the macromolecule, and the coupling of each QMT molecule was carried out independently in the order QMT3, QMT4, QMT7, and QMT8, respectively; the parameters for the Grid-Box were established taking into account the region of the FAD ligand co-crystallized with the enzyme, establishing the following size parameters 60 × 60 × 60 Å and coordinates 22, 10, -16 Å on each axis (X, Y, Z) respectively, centered on the active site of the enzyme with fundamental amino acids coordinates of the active site of the enzyme (Cys53, Cys58 and His461) (Fig. 2) (Beig et al. 2015).

Each coupling provides ten conformations with their respective interaction energies arranged in tables. The results were visualized in ADT and with the help of Discovery Studio (Biovia 2020), which allows generating maps showing the types of interaction between the molecules present and the respective amino acid residues of the enzyme.

The choice of the enzyme trypanothione reductase (TryR) as a molecular target is based on its essential role in the unique redox system of *T. cruzi*, which relies on trypanothione rather than glutathione. TryR is responsible for maintaining this thiol in its reduced form, allowing the parasite to neutralize ROS. Furthermore, previous studies have shown that the inhibition of TryR with low molecular weight compounds directly interferes with redox homeostasis, leading to an intracellular accumulation of ROS and parasite cell death (Irigoin et al. 2008; Krauth-Siegel and Comini 2008). For these reasons, we focused our docking experiments on TryR to evaluate the affinity of the ligands (coordination compounds) selected and explore their potential as inhibitors of this key enzyme. The results regarding the interactions between the coordination complex and the protein are interpreted from a semi-quantitative perspective, useful for identifying potential trends in binding sites and relative affinities, but not as a precise estimation of binding energies or Ki values.

## Results

### QMT3 and QMT8 compounds reduce the mobility and survival of epimastigotes

**Fig. 3:**
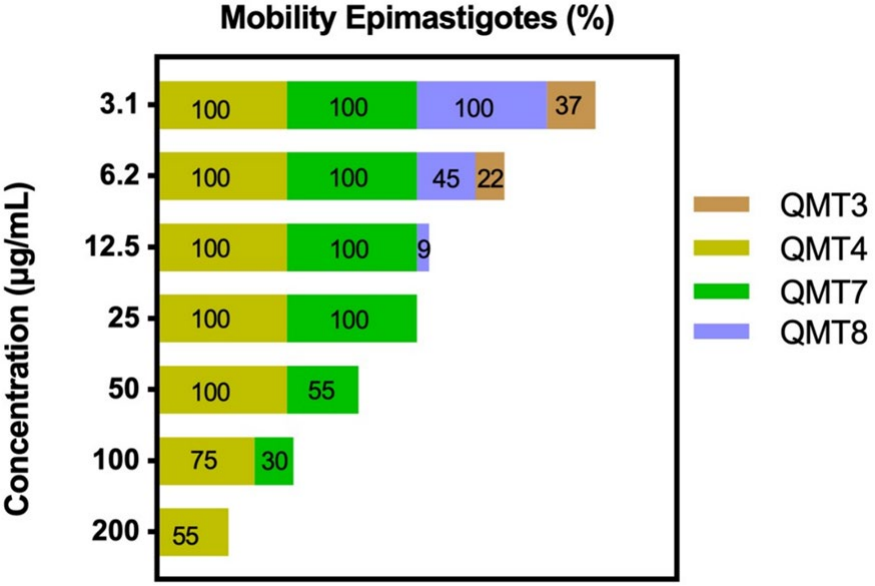
Effect of QMT3, QMT4, QMT7 and QMT8 on epimastigotes mobility. Parasites were incubated with varying concentrations (200 µg/mL to 3.1 µg/mL) from each compound for 24 h (n = 3). Mobility was assessed by optical microscopy and data were compared in relation to the untreated parasites

The activity of the carbene compounds against epimastigotes mobility was evaluated by optical microscopy at concentrations ranging from 200 to 3.1 µg/mL. As depicted in Fig. 3, QMT3 drastically affected parasite mobility, resulting in 78% and 63% reductions at 6.2 and 3.1 µg/mL concentrations, respectively. Remarkably, concentrations exceeding 6.2 µg/mL completely inhibited parasite movement. Similarly, QMT8 decreased this biological property across all concentrations tested, with mobility suppression observed above 12.5 μg/mL. A 91% reduction in epimastigote motility was observed at 12.5 µg/mL and a 45% reduction at 6.2 μg/mL, without detectable effect at 3.1 µg/mL. In contrast, QMT4 and QMT7 had minimal impact on parasite movement, showing decreased epimastigotes mobility only at concentrations above 50 µg/mL for QMT4 and 100 µg/mL for QMT7 (Fig. 3).

**Fig. 4:**
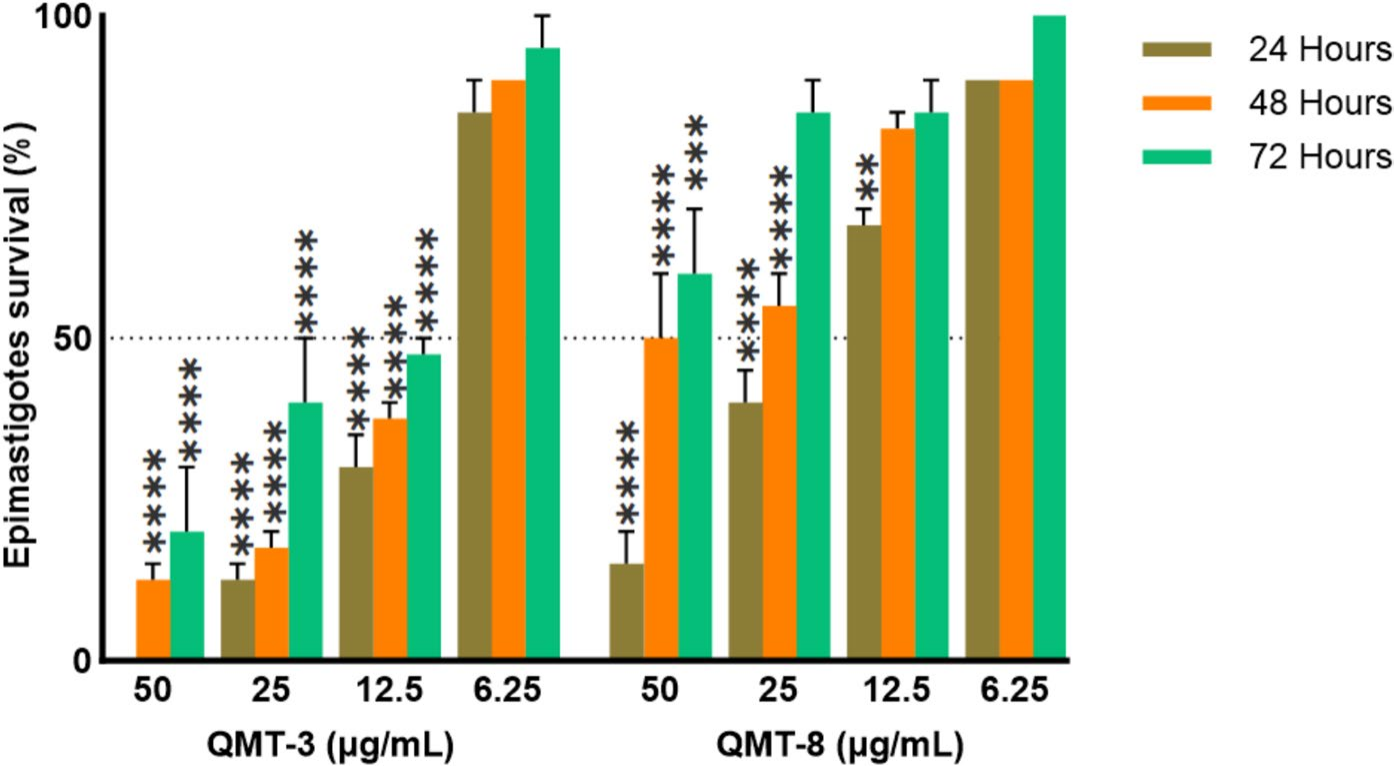
Effect of QMT3 and QMT8 on epimastigotes survival. Epimastigotes were exposed to varying concentrations of QMT3 and QMT8 (200 to 6.2 µg/mL) for 24, 48, and 72 h. Viability was assessed using the trypan blue dye exclusion assay. Each bar represents the mean ± standard error of three independent experiments. Statistical significance was determined by one-way ANOVA with multiple comparisons against the untreated group (** p ≤ 0.01, ****p ≤ 0.0001)

All compounds were analyzed for epimastigotes viability using a dye exclusion test. QMT4 and QMT7 compounds showed no cell growth inhibition at concentrations below 200 µg/mL. In contrast, QMT3 and QMT8 exhibited significant reductions in cell viability at concentrations as low as 50 µg/mL (Fig. 4). QMT3 presented the highest effect on parasite survival among the analyzed compounds, exhibiting a dose-dependent reduction in the epimastigotes stage with an IC_50_ value of 10.3 µg/mL (17.2 µM) after 24 h. Similar IC_50_ values were observed for 48 (11.2 µg/mL) (18.7 µM) and 72 h (12.4 µg/mL) (20.7 µM). In addition, QMT8 displayed the second most potent cytotoxic activity, causing a dose-dependent decrease in parasite survival with an IC_50_ value of 21.2 µg/mL (30.1 µM) after 24 h. However, its activity diminished significantly over prolonged periods, with IC_50_ values above 200 µg/mL at 48 and 72 h (Fig. 4). To determine whether the loss of parasite survival was reversible after QMT3 and QMT8 removal, we monitored epimastigote for up to 72 h. Notably, no survival recovery was observed over time with both compounds (Supplementary information). These findings suggest that treatment with QMT3 and QMT8 induces irreversible cell death rather than a transient stress response.

Considering the anti-trypanocidal activity of QMT3 and QMT8, these compounds were evaluated against the no-tumoral Vero cell line using an MTT assay to determine their safe dosage. QMT3 showed a moderate effect on cell viability, with a reduction of 36.4% at the lowest concentration tested (12.5 µg/mL) for 24 h. In addition, this compound exhibited cytotoxic concentration 50 (CC_50_) values of 108.3 µg/mL (324.5 µM) for 24 h, 32.6 µg/mL (103.1 µM) for 48 h, and 28.8 µg/mL (86.3 µM) for 72 h, indicating that the IC_50_ value estimated for QMT3 (10.3 µg/mL) will not reduce the cell host viability below 50% after 24 h.

On the other hand, QMT8 presented lower cytotoxic activity against Vero cells than QMT3. This compound showed a reduction in cell survival of 29.5% at 12.5 µg/mL for 24 h, with CC_50_ values of 131.8 µg/mL (463.9 µM) for 24 h, 95.4 µg/mL (335.7 µM) for 48 h, and 46.5 µg/mL (163.7 µM) for 72 h, suggesting reduced cytotoxicity over longer durations. This behavior was also observed in the parasite assays. Additionally, the selectivity index (SI) was determined for both compounds (Table 1). QMT3 exhibited anti-trypanosomal activity against *T. cruzi* and showed a favorable SI, demonstrating more potent antiparasitic activity than QMT8, which displayed moderate selectivity.

**Table 1:** QMT average IC_50_, CC_50_ and SI values for Vero cell line

Compounds	IC_50_ (µg/mL)	CC_50_ (µg/mL)	SI
QMT3	10.3	108.3	10.51
QMT8	21.2	131.8	6.21

Values represent the mean of 3 determinations obtained in independent experiments performed at 24 h. SI = Selective index values calculated are the ratio CC_50_/IC_50_.

### QMT3 and QMT8 mainly induce late apoptotic-like and/or necrotic cell death in epimastigotes

Based on the cytotoxic effects observed in epimastigotes treated with QMT3 and QMT8, annexin V/PI labeling analysis was conducted on parasites exposed to these compounds at concentrations ranging from 50 to 6.2 µg/mL for 24 h. In untreated cells, most were negatively stained for annexin V and PI, showing 99.8% as live cells, 0.1% in early apoptosis-like cell death, 0.1% of cells in late apoptotic-like and/or necrotic cell death, and 0.0% in necrosis.

**Fig. 5:**
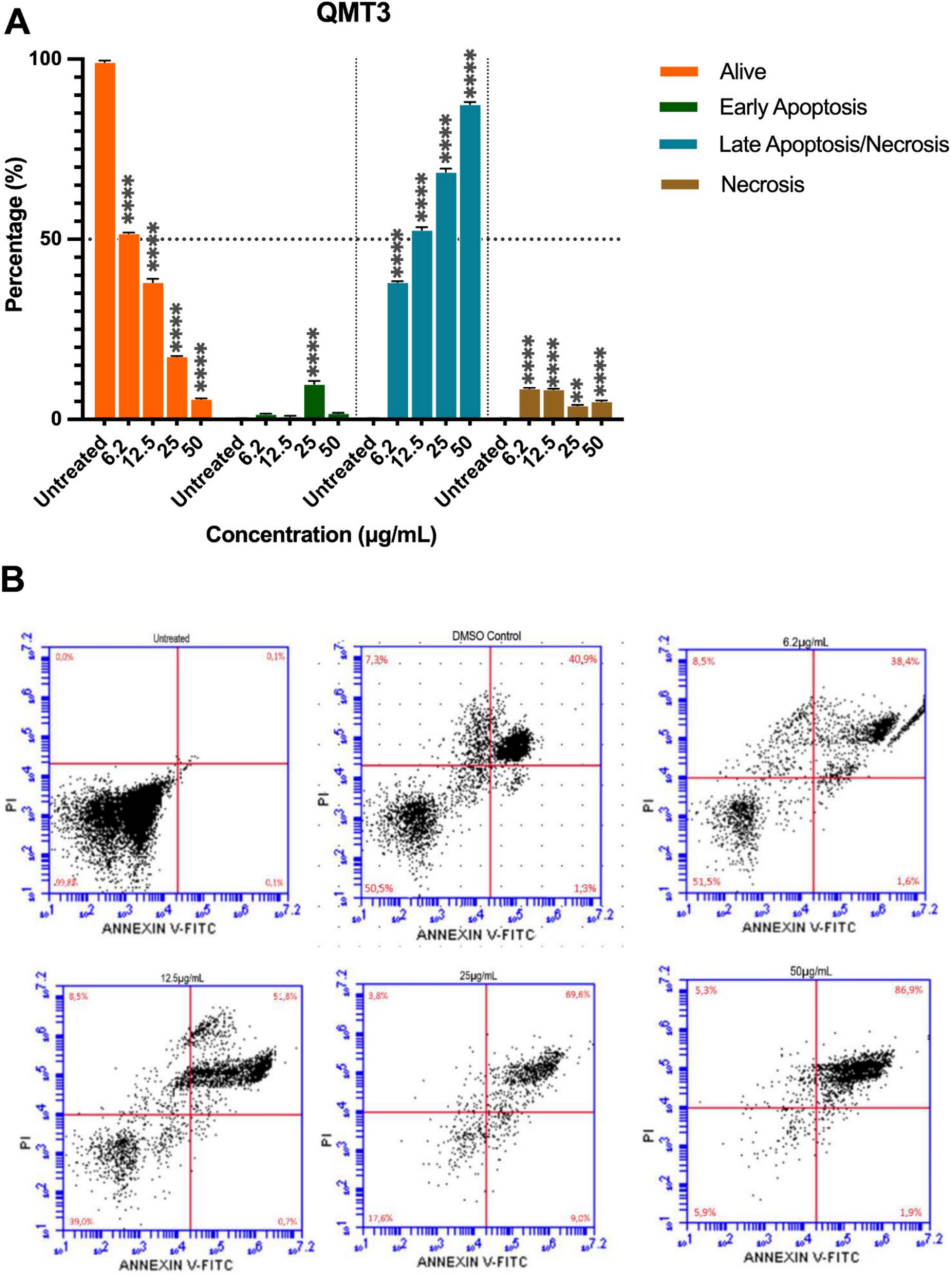
Evaluation of QMT3 on epimastigotes cell death after 24 h of treatment. **A** Epimastigotes were labeled with Annexin V and propidium iodide (PI) after 24 h of exposure to QMT3 at different concentrations (50 to 6.2 µg/mL). The percentage of live cells, necrotic cells, and those in early and late apoptosis-like cell death are displayed. Parasites without treatment were used as a negative control, while 96% DMSO served as a positive control for the induction of cell death. Each bar represents the mean ± standard error of three independent experiments. Statistical significance was determined by one-way ANOVA with multiple comparisons against the untreated group (**p ≤ 0.01, ****p ≤ 0.001). **B** Representative plots of each experimental condition. Percentages in each panel indicate early apoptotic cells (lower right quadrant), late apoptotic cells (upper right quadrant), and necrotic cells (upper left quadrant)

After 24 h of treatment with QMT3, parasites exhibited a significant reduction in the percentage of live cells, accompanied by a substantial increase in late apoptotic-like and/or necrotic cell death compared to untreated parasites across all concentrations analyzed (p < 0.0001) (Fig. 5). In addition, the percentage of parasites in a necrotic stage, without PS exposure, remained around 5% at all tested concentrations. Epimastigotes treated with QMT3 at 12.5 µg/mL showed a 61% decrease in live cells, 51.8% in late apoptotic-like and/or necrotic cell death, and 0.7% in early apoptosis-like cell death. These results indicate that at the estimated IC_50_ of QMT3 (10.3 μg/mL), most cells are undergoing late apoptotic-like and/or necrotic cell death.

**Fig. 6:**
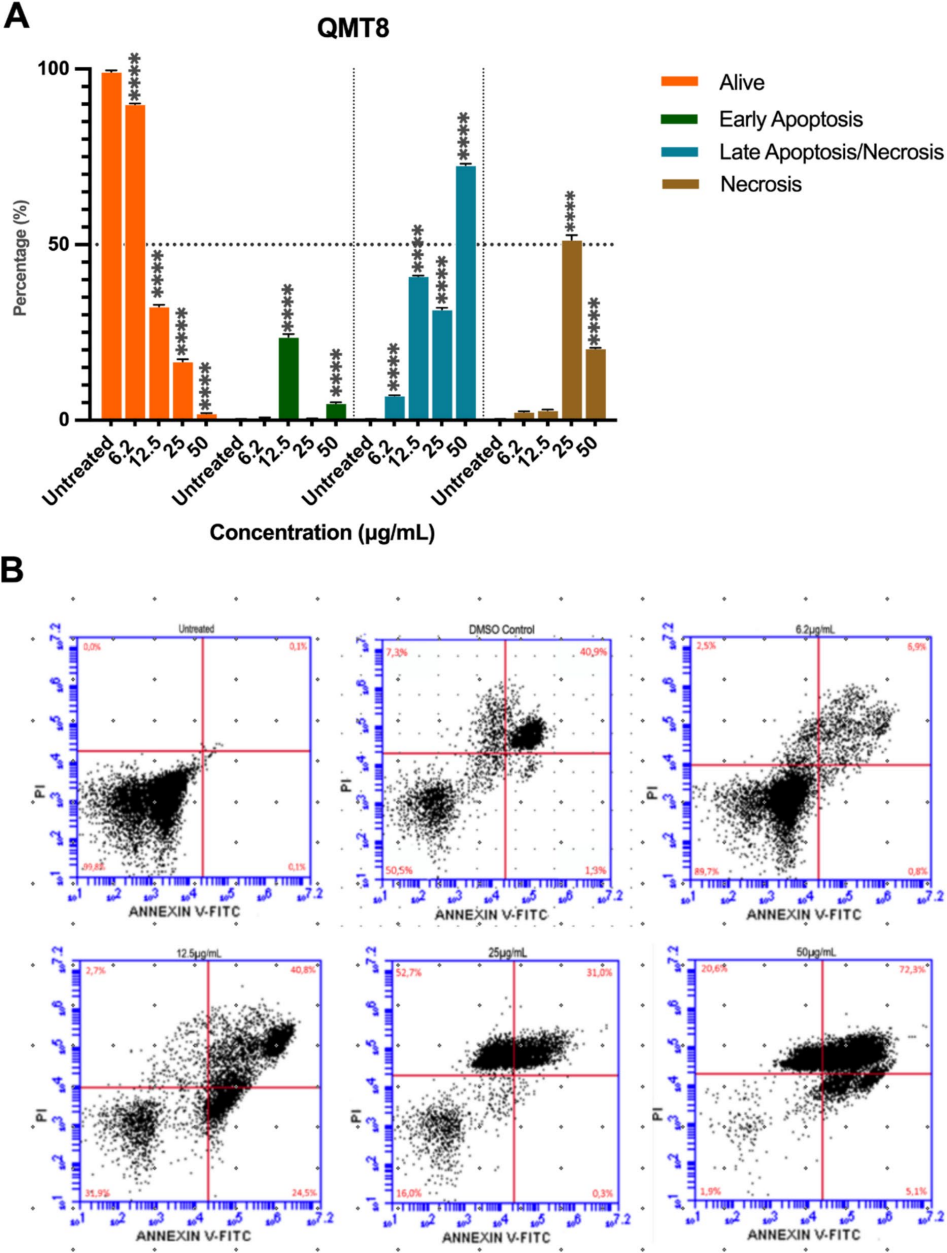
Evaluation of QMT8 on epimastigotes cell death after 24 h of treatment. **A** Epimastigotes were labeled with Annexin V and propidium iodide (PI) after 24 h in contact with QMT3 at different concentrations (50 to 6.2 µg/mL). The percentage of live cells, necrotic cells, with early apoptosis-like cell death and late apoptosis-like cell death is shown. Parasites without treatment were used as a negative control, while 96% DMSO served as a positive control for the induction of cell death. Each bar represents the mean ± standard error of three independent experiments. Statistical significance was determined by one-way ANOVA with multiple comparisons against the untreated group (****p ≤ 0.001). **B** Representative plots of each experimental condition. Percentages in each panel indicate early apoptotic cells (lower right quadrant), late apoptotic cells (upper right quadrant), and necrotic cells (upper left quadrant)

Parasites treated with QMT8 for 24 h revealed a significant reduction in live cells, particularly at the highest tested doses of the compound (Fig. 6). Treatment with QMT8 at 50 µg/mL drastically decreased the percentage of live cells to 2%, while the proportion of parasites in late apoptotic-like and/or necrotic cell death rose to 72.3%. Additionally, necrosis, without PS exposure, accounted for 20.6%, and early apoptosis-like cell death represented 5,1%. For the 25 µg/mL concentration, most cells were undergoing necrosis (52.7%) and late apoptotic-like and/or necrotic cell death (31%), with only 16% remaining alive. At a 12.5 µg/mL concentration, 40.8% of epimastigotes exhibited late apoptotic-like and/or necrotic cell death, with 31.9% remaining alive, 24.5% in early apoptosis-like cell death, and less than 3% in a necrotic stage. Interestingly, at the lowest concentration evaluated (6.2 µg/mL) 90% of the parasites were alive, followed by 6.9% in late apoptotic-like and/or necrotic cell death and less than 3% in a necrotic state.

### ROS production in parasites exposed to QMT3 and QMT8

**Fig. 7:**
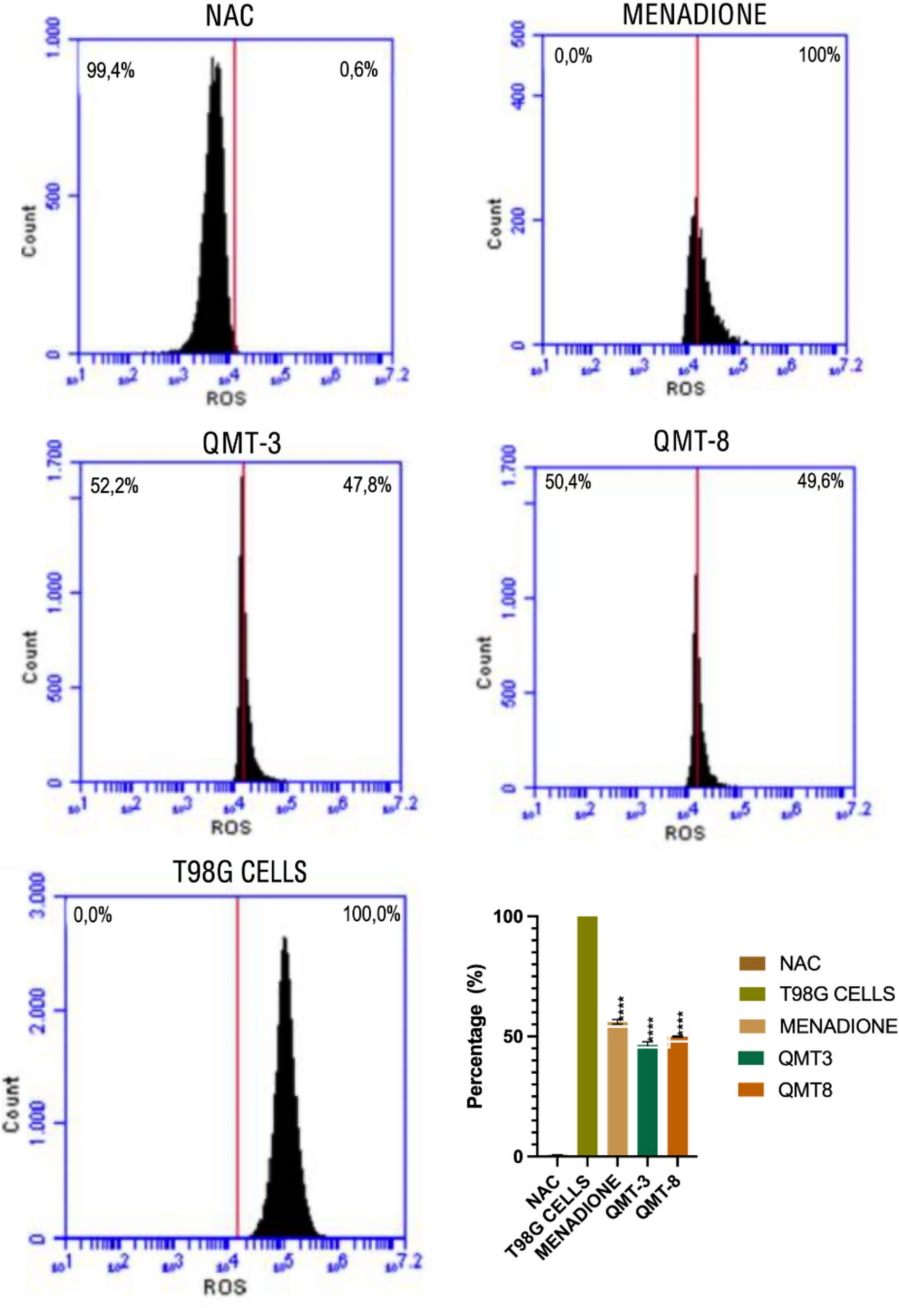
Intracellular ROS levels in *T. cruzi* epimastigotes exposed to QMT3 and QMT8. ROS levels were assessed by flow cytometry after 24 h of incubation with the IC₅₀ concentrations of QMT3 and QM-8. N-acetylcysteine (NAC) was used at 5 mM as a negative control, while menadione (25 µg/mL) served as a positive control for ROS induction in *T. cruzi*. Human T98G cell line was treated with pyocyanin (200 µM) as an additional control to validate assay specificity. Data represent the mean ± standard error of three independent experiments. Statistical analysis was performed using one-way ANOVA with multiple comparisons relative to the untreated control group (****p ≤ 0.0001)

**Fig. 8:**
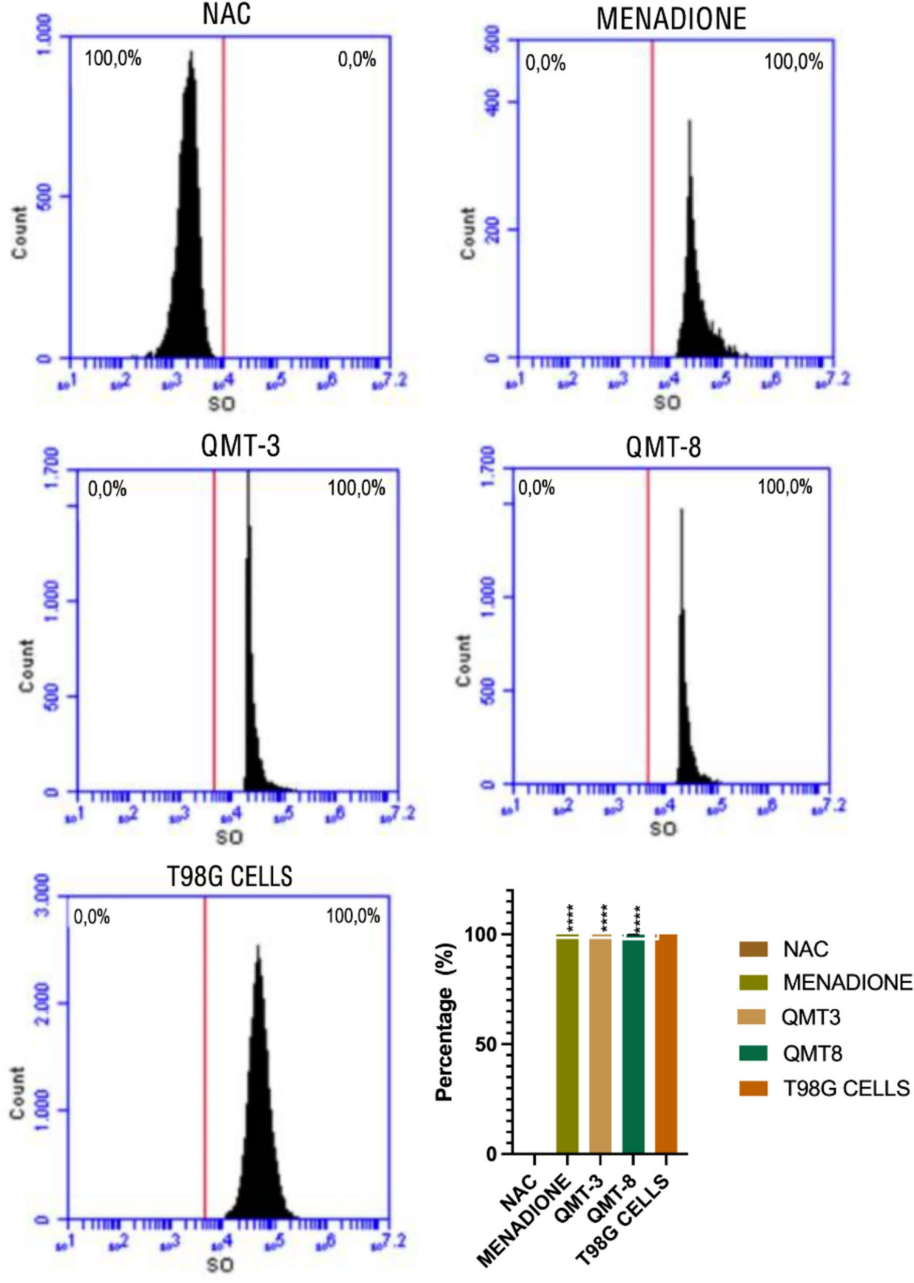
Intracellular SO levels in *T. cruzi* epimastigotes exposed to QMT3 and QMT8. SO levels were assessed by flow cytometry after 24 h of incubation with the IC₅₀ concentrations of QMT3 and QM-8. N-acetylcysteine (NAC) was used at 5 mM as a negative control, while menadione (25 µg/mL) served as a positive control for ROS induction in *T. cruzi*. Human T98G cell line was treated with pyocyanin (200 µM) as an additional control to validate assay specificity. Data represent the mean ± standard error of three independent experiments. Statistical analysis was performed using one-way ANOVA with multiple comparisons relative to the untreated control group (****p ≤ 0.0001)

Given the anti-trypanocidal activity exhibited by QMT3 and QMT8, the levels of ROS and SO were assessed in epimastigotes after 24-h exposure to the IC₅₀ concentrations of each carbene. Parasites treated with NAC displayed low ROS and SO levels, whereas those exposed to menadione, a known ROS inducer in *T. cruzi*, showed increases of 55% in ROS and 100% in SO levels (Fig. 7 and 8). Additionally, a mammalian cell line treated with pyocyanin was included as a supplementary positive control due to its high susceptibility to ROS production, further validating the reliability of the intracellular ROS/SO assay. This control effectively induced both ROS and SO accumulation, confirming proper labeling function and system responsiveness. Notably, QMT3 and QMT8 treatment resulted in a significant increase in ROS (~ 49%, p ≤ 0.0001) and SO (100%, p ≤ 0.0001), indicating that both compounds impair the oxidative stress regulation system of *T. cruzi* (Figs. 7, 8).

#### Docking between NHC and TryR of T. cruzi

The Table 2 presents the results of docking simulations performed using AutoDock4 version 1.5.2. The Lamarckian genetic algorithm was employed for generating the output file. The docking experiments yielded the following binding free energy values, expressed in kcal/mol.

**Table 2:** Binding energies Coupling (∆G) in kcal/mol for 10 docked poses of the selected compounds with the active site region for the enzyme 1AOG

Conformation	QMT3	QMT4	QMT7	QMT8
1	-6.12	-10.42	-6.63	-7.23
2	-7.84	-10.48	-6.63	-7.23
3	-6.73	-10.14	-6.63	-7.01
4	-6.70	-10.53	-6.63	-7.25
5	-6.68	-10.18	-6.63	-7.08
6	-6.35	-9.52	-6.63	-7.25
7	-6.41	-10.50	-6.63	-6.49
8	-6.32	-10.50	-6.63	-6.49
9	-7.77	-10.05	-6.63	-7.25
10	-7.79	-10.02	-6.63	-6.49

Underlined values indicate the best interaction energies for each compound.

The following figure shows the result of the docking between the complexes under study:

The Ki value was calculated from the coupling energy using the standard AutoDock formula; this is an estimated theoretical value, not an experimental one.

The ligands were subjected to molecular docking after the preparation described previously. The analysis of the results was based on the binding free energy and the inhibition constant (Ki) of the complex formed between the target protein and the best conformations obtained (Table 3). The compounds studied presented binding energies that ranged between -6.63 kcal/mol (QMT7) and -10.53 kcal/mol (QMT4), with inhibition constants calculated between 1.81 µg/mL and 19.04 µg/mL, respectively. It is important to emphasize that the binding energy values, and inhibition constants (Ki) presented in this study are theoretical estimates obtained through computational models. Therefore, their interpretative value is relative and indicative and should not be considered equivalent to direct experimental data. This limitation is inherent to the docking method employed and has been acknowledged in other similar studies (Ortega-Carrasco et al. 2014).

**Table 3:** Better in silico values of molecular docking between 1AOG and the QMT3, QMT4, QMT7 and QMT8

Molecule	Nature of the interaction	Molecular interaction	Distance (Å)	Coupling bond energy (∆G) (Kcal/mol)	Estimated inhibition constant (Ki) (μM)
QMT3	Hydrophobic	Met 333, Leu 334, Cys 58		-7.84	1.81
	Polar	Thr 52, Ser 163, Thr 335			
QMT4	Hydrogen bond	Ag-Glu 19	2.87	-10.53	19.04
	Hydrophobic	Trp 22, Ile 339			
	Polar	Asn 23			
QMT7	Hydrophobic	Leu 18, Pro 336, Ile 339, Tyr 111, Cys 53, Val 54, Val 59		-6.63	13.86
	Polar	Thr 335, Ser 15			
QMT8	Hydrophobic	Ile 11, Ala 13, Val 37, Ile 35, Ala 160		-7.25	4.86
	Polar	Ser 47, Thr 52, Ser 161			

The low Ki values for QMT3 and QMT8 suggest a higher binding affinity with the enzyme compared to QMT4 and QMT7. The compound QMT4 exhibits the best binding energy; however, it has the highest value for the Ki constant, which could influence its ability to inhibit the pharmacological target under study. On the other hand, compound QMT3 presents the second-best binding energy (-7.84 kcal/mol) and the lowest Ki value, which allows establishing a positive correlation between the binding energy and the inhibition constant.

**Fig. 9:**
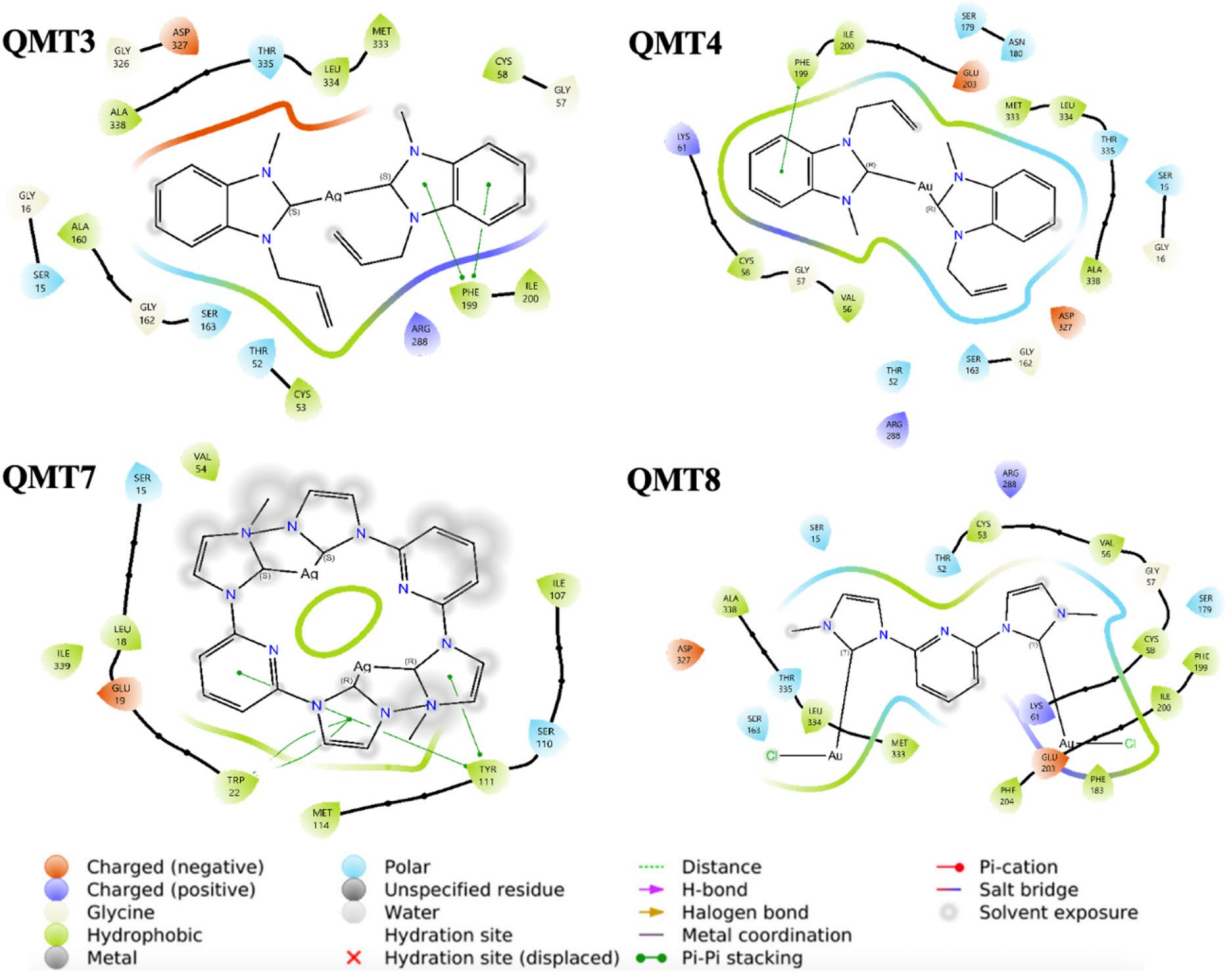
The 2D interactions of the TryR protein with the compound QMT3, QMT4, QMT7 and QMT8 in the best energy conformations, with the respective residues and their types of interaction with the ligands

The hydrophobic, polar and hydrogen bond interactions between the ligands and the amino acids of 1AOG were also analyzed in 3D and 2D, using the AutoDockTools 1.5.7 and Maestro 14.1 programs. The QMT3-1AOG complex showed hydrophobic and polar interactions with residues Met333, Leu334, Cys58, Thr52, Ser163 and Thr335 (Fig. 9). Although hydrogen bonds were not observed, interactions with Cys58 and Thr52 may be relevant, since these residues are part of the catalytic site of the enzyme (Zhang et al. 1996).

For compound QMT4, hydrophobic and polar interactions were identified with amino acids Trp22, Ile339 and Asn23, as well as the formation of a hydrogen bond between silver (Ag) and Glu19, which could favor the possible inhibition of the enzyme (Fig. 9). In the case of QMT7 and QMT8, hydrogen bonds were not observed with the protein residues; however, they present hydrophobic and polar interactions with amino acids that participate in the catalytic activity and stabilization of the enzyme, such as Thr52 and Cys53.

## Discussion

There is a need for new agents to treat Chagas disease, and NHC compounds, particularly those linked to silver or gold, have demonstrated significant anticancer (Haque et al. 2015; Zou et al. 2018), antimicrobial (Estrada-Ortiz et al. 2017; Gil-Moles et al. 2023; Kascatan-Nebioglu et al. 2006), and anti-parasitic activities (Ataş et al. 2024; Hemmert et al. 2013; Rosa et al. 2022; Winter et al. 2017; Zhang et al. 2018). Therefore, we evaluated the activity of four NHC-type compounds, linked to silver or gold, against *T. cruzi*.

Among the compounds tested, QMT3 and QMT8 showed the most significant effects on the mobility of the epimastigote form of *T. cruzi*. QMT3 exhibited the most pronounced impact on parasite survival, with a dose-dependent reduction in the epimastigote stage, showing an IC_50_ value of 10.3 µg/mL at 24 h. In addition, QMT8 exhibited the second most potent cytotoxic activity, also reducing parasite survival in a dose-dependent manner, with an IC_50_ value of 21.2 µg/mL at the same time point. Notably, both compounds had minimal effects on the viability of mammalian cells, although QMT8 was less cytotoxic to mammalian cells than QMT3 after short exposure times. These findings indicate that QMT3 and QMT8 exhibited the highest trypanocidal activity among the four synthesized compounds, with good specificity for *T. cruzi* and low IC_50_ values. QMT3, in particular, demonstrated more consistent activity over time and a lower IC_50_ value compared to QMT8.

Importantly, both QMT3 and QMT8 induced complete and irreversible parasite death within 24 h of treatment. Parasites were unable to recover viability after compound removal, even in the absence of further exposure. This property is particularly valuable for treating Chagas disease, where rapid and definitive parasite clearance is essential to prevent recrudescence and to reduce host toxicity associated with prolonged drug administration. Therefore, despite the decreasing SI over time for QMT3, its short-term potent and irreversible action supports its potential as a promising therapeutic candidate. Future formulations or dosing strategies could be optimized to exploit this early-stage effect while mitigating host toxicity.

Interestingly, beyond their cytotoxic properties, QMT3 induced a significant increase (~ 52%) in late apoptotic-like and/or necrotic cell death compared to untreated parasites after 24 h of treatment. This effect was accompanied by a marked elevation in ROS levels, particularly SO. For QMT8, the reduction in live cells observed at 24 h was associated with an increase in both early and late apoptotic-like and/or necrotic cell death. At the highest concentrations tested, a small proportion of necrotic cells lacking PS exposure was also detected. Similar to QMT3, QMT8 triggered a significant rise in ROS levels, again with notably high levels of SO. Taken together, these findings suggest that both compounds disrupt parasite survival pathways, likely through dysregulation oxidative stress response system of *T. cruzi*.

Although research on metal-NHC complexes in trypanosomatids is limited, several studies have been conducted in cancer cells. In cancer models, silver NHC complexes have been shown to activate apoptosis death independent of caspases (Doğan Ulu et al. 2024; Iqbal et al. 2015; Li et al. 2014), targeting mitochondria and promoting the translocation of apoptosis inducing factor (AIF) and caspase-12 from the mitochondria to the endoplasmic reticulum (ER) and nucleus, respectively (Eloy et al. 2012). Gold NHC compounds, on the other hand, have been associated with mitochondrial cell death due to their accumulation in this organelle, which depends on mitochondrial membrane potential, ROS production, cytochrome c release, ER stress, lysosomal damage, and caspase activation, thereby inducing apoptosis (Tang et al. 2025; Zhao et al. 2024).

In trypanosomatids, NHC complexes with different metal elements such as gold (Winter et al. 2017), iridium, and rhodium (Simpson et al. 2013) have shown trypanocidal properties. Evidence suggests that metal-based NHC complexes target organelles such as the cytoskeleton (Winter et al. 2017), nucleus (Rodriguez-Cabezas et al. 2001), and membranes (Minori et al. 2020; Rosa et al. 2022). Although the molecular targets of these compounds are not fully defined, in the case of complexes with iridium and rhodium, their mechanism of action has been attributed to the inhibition of thioredoxin reductase (TrxR) a protein involved in the antioxidant defense of the parasite (Winter et al. 2017) but are not related with the glutathione reductase (Hickey et al. 2008; Rackham et al. 2007). It is noteworthy that QMT8 is a NHC compound coordinated with two gold atoms, whereas QMT3 is coordinated with a single silver atom. These structural differences may underlie distinct mechanisms of parasite death.

Compounds complexed with gold or silver have been shown to influence cellular redox mechanisms, primarily through interactions with thiol- and selenium-containing proteins (Berners-Price and Filipovska 2011). Among these, TrxR is the most extensively studied molecular target and is present in various cell types, including cancer cells and HIV-infected models (Sánchez et al. 2016). Gold complexes inhibit TrxR by forming covalent bonds with the enzyme’s selenium residues, leading to conformational changes that disrupt its redox function (Nobili et al. 2010). This mechanism is particularly relevant in the context of parasitic infections, as redox-regulating proteins are essential for the survival of pathogens under oxidative stress.

In trypanosomes, malaria parasites, and schistosomes, thiol- and selenoproteins such as trypanothione reductase, tryparedoxin, glutathione peroxidase, selenoprotein P, and selenium-containing TrxRs have been identified as potential drug targets (Fricker et al. 2008; Krauth-Siegel et al. 2005). These proteins are critical for maintaining redox homeostasis and protecting the parasite from oxidative bursts generated by host immune responses. Therefore, the antiparasitic activity of NHC-metal complexes may be mediated, at least in part, by interference with this redox system, highlighting their potential as selective therapeutic agents against trypanosomatids.

Trypanosomatids possess a distinct redox metabolism that relies on the thiol-polyamine compound trypanothione and the flavoenzyme TryR (Krauth-Siegel et al. 2005). The enzyme reduces oxidized trypanothione to its reduced form using electrons from NADPH. This reaction is essential for maintaining a high ratio of reduced trypanothione, used in antioxidant processes (Collins and Donadio 2011). Notably, TryR is distinct from glutathione reductase found in most other eukaryotic cells. While both enzymes reduce disulfide bonds, TryR specifically reduces trypanothione and is adapted to the unique biochemical environment of trypanosomatids.

Our molecular docking analysis suggests that QMT3, QMT4, and QMT8 may interact with the active site of TryR, potentially competing with its natural ligand and binding to amino acid residues known to be important for its redox activity. In contrast, QMT7, due to its larger molecular volume, could not access the active site and instead interacted with the enzyme’s periphery, suggesting a possible allosteric inhibitory mechanism. However, given the known limitations of in silico modeling for metal-containing compounds, these docking predictions should be considered preliminary. The electronic configuration and geometry of silver and gold atoms may influence ligand orientation and binding affinity. Consequently, further experimental validation, such as co-crystallization and structural studies, is essential to confirm these theoretical findings.

This study highlights the promising trypanocidal potential of silver- and gold-based NHC complexes as novel candidates for the treatment of Chagas disease. Among the four synthesized compounds, QMT3 and QMT8 emerged as the most effective, demonstrating significant antiparasitic activity against *T. cruzi*, with QMT3 exhibiting superior potency, sustained activity over time, and a lower IC₅₀. Both compounds induced rapid and irreversible parasite death and showed selective toxicity, causing minimal harm to mammalian cells at therapeutic concentrations. Our results suggest that QMT3 and QMT8 trigger apoptosis-like and necrotic pathways in the parasite, potentially through disruption of redox homeostasis. This was supported by increased ROS and SO production. Molecular docking studies further revealed strong interactions of QMT3 and QMT8 with the active site of TryR, a key enzyme in the antioxidant defense system of the parasite. These interactions, particularly the favorable binding energies and predicted inhibition constants, reinforce the hypothesis that these metal–NHC compounds act, at least in part, by targeting TryR and impairing the redox balance essential for parasite survival.

## Supplementary Information

Below is the link to the electronic supplementary material.

Supplementary file1 (PDF 1376 KB)

## Data Availability

Data is provided within the manuscript.
