# Supplementary material for: Targeting Trypanosoma cruzi with silver and gold-based N-heterocyclic carbene complexes: insights into parasite death and trypanothione reductase interaction
Source: Biometals. 2025 Aug 14;38(6):1795–813. doi: 10.1007/s10534-025-00731-4 (PMC12686042; doi:10.1007/s10534-025-00731-4)
Supplement: Supplementary file 1 — Supplementary file1 (PDF 1376 KB) [file 10534_2025_731_MOESM1_ESM.pdf]

Supplementary information

Targeting *Trypanosoma cruzi* with silver and gold-based N- heterocyclic carbene complexes: insights into parasite death and trypanothione reductase interaction

Yuly Bernal[1], Angie Melo Marquez[1], Hector Rafael Rangel[2], Maria Cristina Goite[3], Pedro Noguera[3], Franmerly Fuentes[3], Rubén Machado[3], William Castro[3], Vaneza Paola Lorett Velasquéz[4], Cristian Buendia-Atencio[1], Eduvan Valencia Cristancho[5], Anny Karely Rodriguez[1], Silvio Lopez-Pazos[1], Monica Losada-Barragán[1*]

[1] Facultad de Ciencias, Universidad Antonio Nariño, Bogotá D.C., Colombia.

[2] Venezuelan Institute for Scientific Research | IVIC · Laboratorio de Virología Molecular, Caracas, Venezuela.

[3] Instituto Venezolano de Investigaciones Científicas | IVIC · Centro de Química, Instituto Venezolano de Investigaciones Científicas (IVIC), Caracas 1020-A, Venezuela.

[4] Facultad de Medicina y Ciencias de la Salud, Universidad Militar Nueva Granada, Bogotá D.C., Colombia.

[5] Área de Ciencias Naturales, Secretaría de Educación Distrital de Bogotá, Bogotá D.C., Colombia.

*Correspondence: Dra. Monica Losada Barragán. Universidad Antonio Nariño-Sede Circunvalar. Cra. 3 este # 47A - 15, Bogotá, Colombia. E-mail: monica.losada@uan.edu.co

1. Synthetic route for the complexes QMT3 and QMT4

The synthesis of QMT3 and QMT4 was reported previously by Mohammed Z. Ghdhayeb, Rosenani A. Haque, Srinivasa Budagumpi, Journal of Organometallic Chemistry, 757, 2014, 42-50,ISSN 0022-328X, https://doi.org/10.1016/j.jorganchem.2014.01.038.

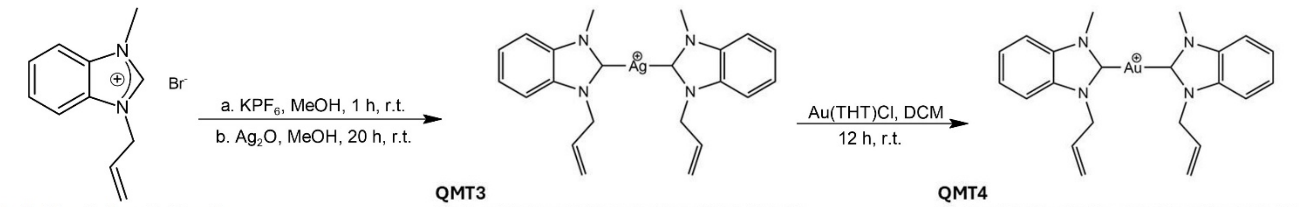

1-allyl-3methylbenzimidazolin-2-yliden-1yl) bromide

QMT3
Bis(1-allyl-3methylbenzimidazolium)silver(I) hexafluorophosphate

QMT4
Bis(1-allyl-3methylbenzimidazolium)gold(I) hexafluorophosphate

2. Synthetic route for the complexes QMT7 and QMT8

The synthesis of QMT7 and QMT8 was reported previously by Oriel Sánchez, Sorenlis González, Ángel R. Higuera-Padilla, Yokoy León, David Coll, Mercedes Fernández, Peter Taylor, Izaskun Urdanibia, Héctor R. Rangel, Joseph T. Ortega, William Castro, María Cristina Goite, Polyhedron, 110, 2016, 14-23, ISSN 0277-5387, https://doi.org/10.1016/j.poly.2016.02.012.

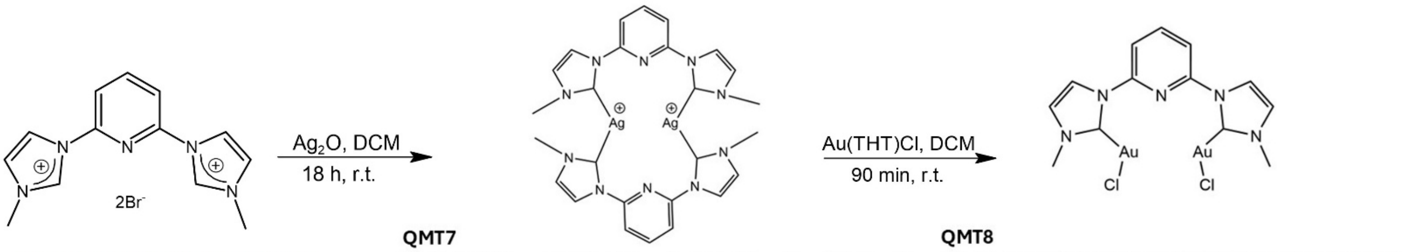

2,6-bis(3-methylimidazolin-2-yliden-1-yl)pyridine dibromide

QMT7
Bis(1,1 -(2,6-pyridyl)-3,3-dimethyldiimidazolium)disilver(I) bromide

QMT8
1,1 -(2,6 pyridyl)-3,3 -dimethyldiimidazolium)dioro(I) bromide

3. ^{1}H NMR spectrum of QMT3 (600 MHz, DMSO-d_6, 298 K).

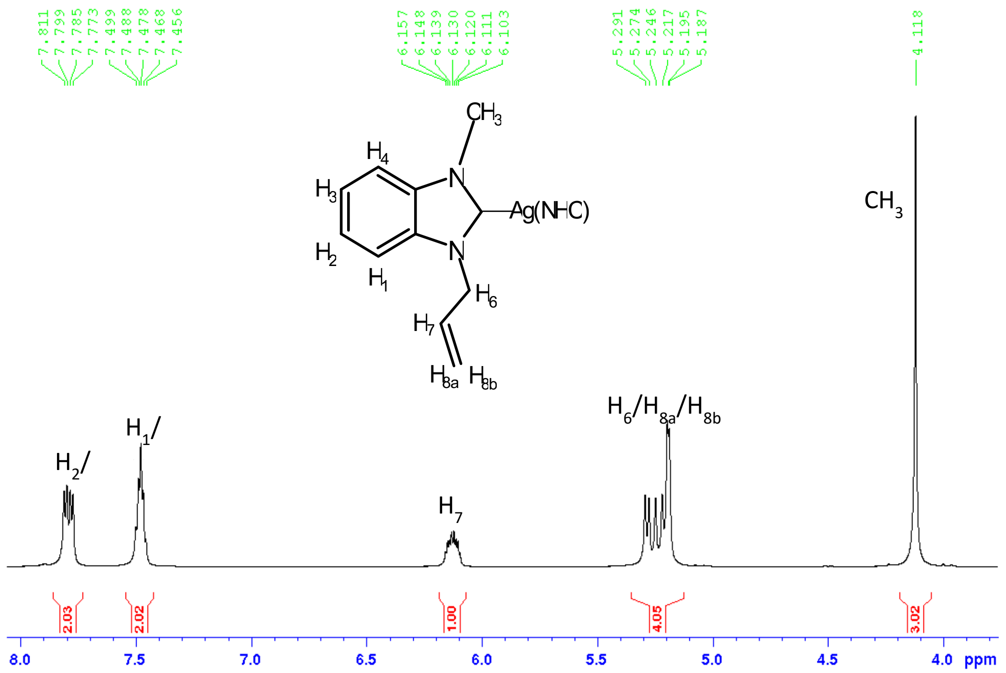

4. 1H NMR spectrum of QMT4 (600 MHz, DMSO-d6, 298 K).

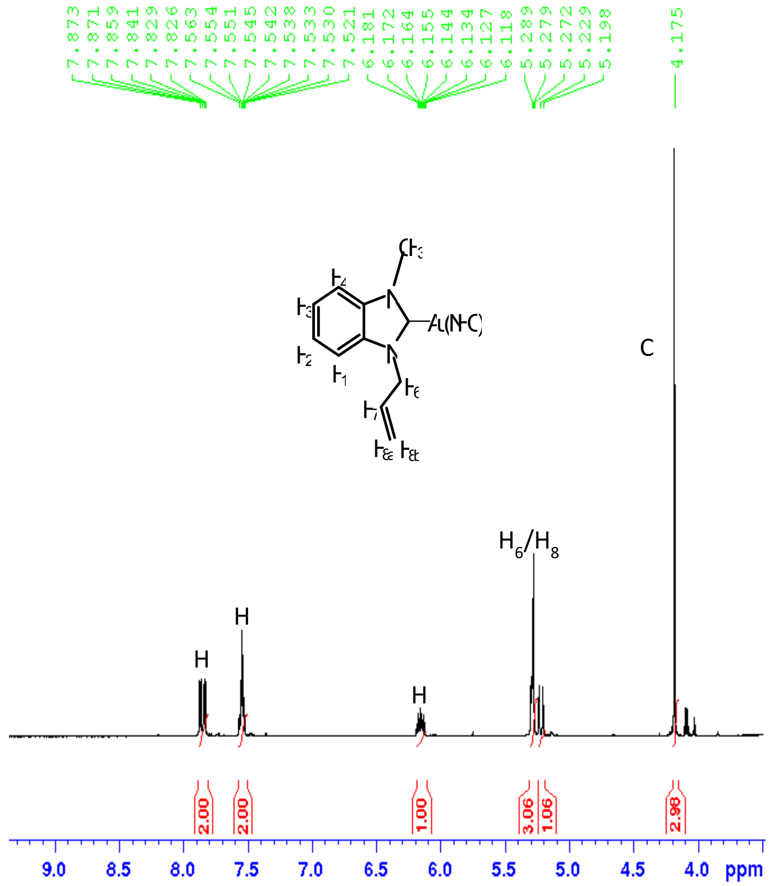

5. ^{1}H NMR spectrum of QMT7: 300 MHz, DMSO-d$_6$ a) at 298 K b) at 353 K

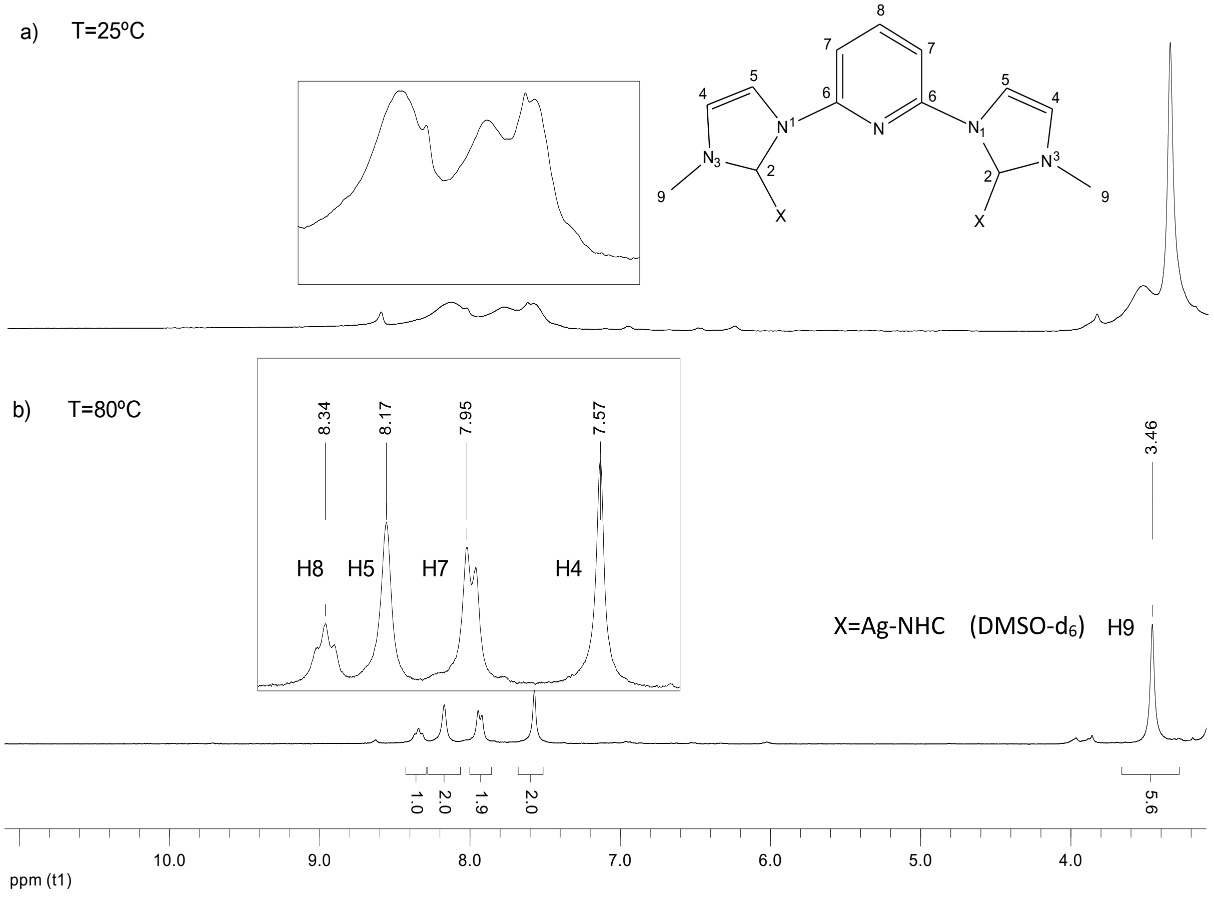

6. ^{1}H NMR spectrum of QMT8: 300 MHz, DMSO-d_6 at 298 K.

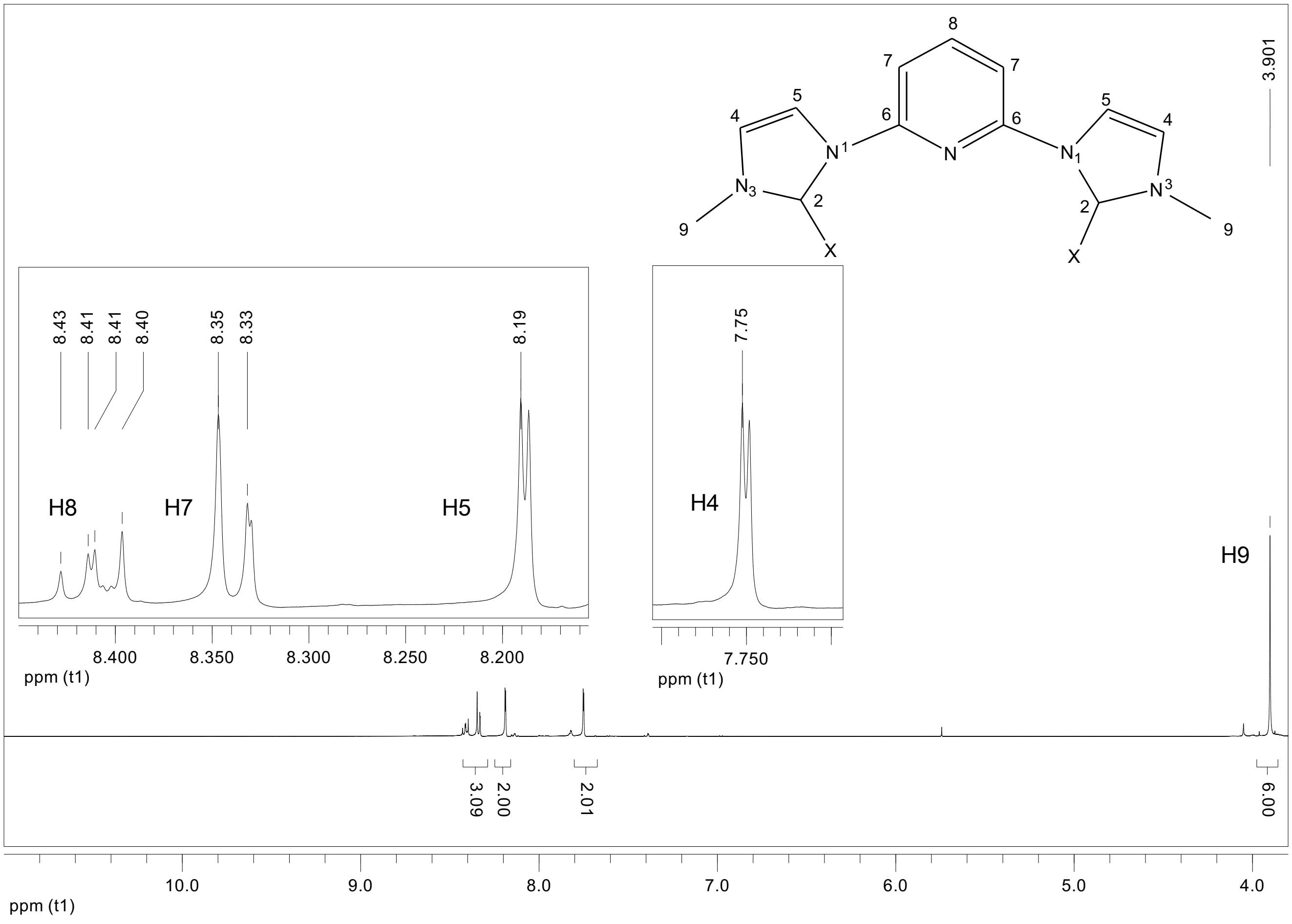

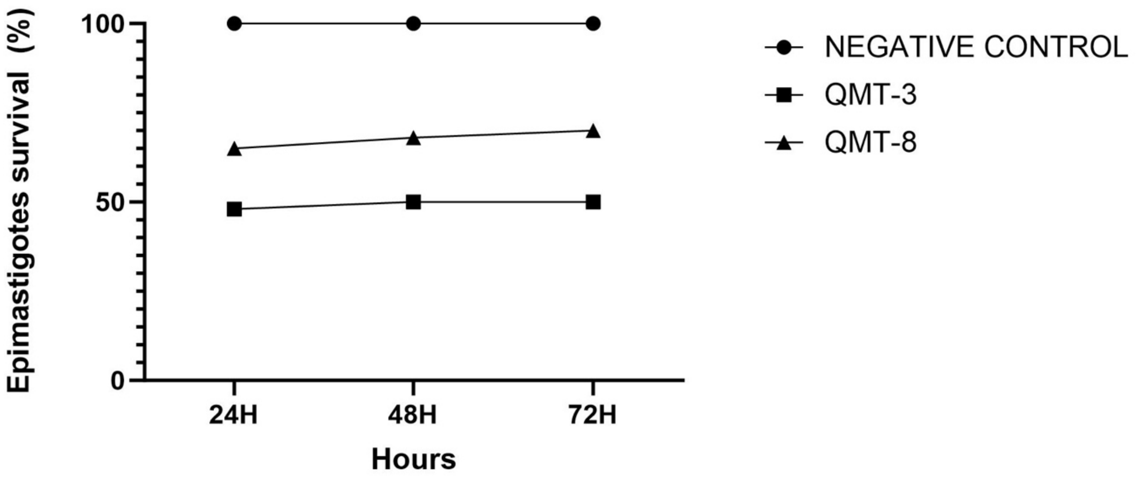

Supplementary figure 1. Effect of QMT3 and QMT8 on epimastigote survival after washing compounds. Epimastigotes were exposed to the IC_{50} concentration of QMT3 and QMT8 for 24 hours, then washed three times with phosphate-buffered saline (PBS) and incubated for up to 72 hours. Parasite survival was evaluated every 24 hours using optical microscopy. Each symbol represents the mean ± standard error of three independent experiments. Statistical significance was determined by one-way ANOVA with multiple comparisons against the untreated group (****$p \leq 0.0001$).
